# Are home-based exercises effective to reduce blood pressure in hypertensive adults? A systematic review

**DOI:** 10.1186/s40885-022-00211-8

**Published:** 2022-09-15

**Authors:** Gabriel Dias Rodrigues, Ligia Soares Lima, Nicole Cristine Simões da Silva, Paula Gomes Lopes Telles, Teresa Mell da Mota Silva Rocha, Victor Quintella de Aragão Porto, Viviane Veloso Cardoso, Pedro Paulo da Silva Soares

**Affiliations:** 1 Laboratory of Experimental and Applied Exercise Physiology, Department of Physiology and Pharmacology, Fluminense Federal University, Niterói, Brazil; 2 National Institute for Science & Technology - INCT Physical (In)Activity & Exercise, CNPq, Niterói, Brazil

**Keywords:** Hypertension, Breathing Exercises, Endurance Training, Resistance Training, Social isolation

## Abstract

Sedentarism and chronic non-communicable diseases have been a worldwide health problem that is drastically exacerbated by the COVID-19 pandemic social impacts. Home-based exercises are widely encouraged during social isolation to counterbalance the physical inactive impacts. Although, in the context of hypertension, are home-based exercises effective in blood pressure controlling? Our objective is to conduct a systematic review of high-quality controlled trials comparing the possible effects of different types of home-based exercises in hypertensive patients. The literature search was carried out in three scientific databases: Medline, Europe PMC, and Lilacs. Articles were included following three criteria: analyzing the effect of home-based exercise programs on blood pressure in treated and untreated hypertensive patients; exercises must perform at home and on the frequency, intensity, time, and type (FITT) principle, and the articles were published in English. From the qualitative analysis of 27 original trials screened through 451 identified studies, the main results are the following: 1) both endurance, isometric strength, and respiratory home-based exercise programs were efficient to decrease blood pressure in hypertensive patients; 2) differences in methodological approaches regarding FITT components, distinct blood pressure values at baseline and specific underlying mechanisms must be considered as a potential bias of each home-based interventions. In conclusion, endurance, isometric strength, and breathing home-based programs seems to be effective to reduce blood pressure in hypertensive patients. However, further randomized controlled trials and mechanistic studies must be performing to guide evidence-based recommendations of home-based exercises as antihypertensive therapy.

## Background

Hypertension remains in the leadership of the causes of deaths globally (> 10.4 million deaths per year). Although billion people worldwide are hypertensive, less than 1 in 5 people have controlled blood pressure [1]. High blood pressure may cause heart damages through the hardening of arteries, decreasing blood flow, and oxygen perfusion to the heart muscle and other tissues. Hypertension is considered as one of the main risk factors for cardiovascular diseases, among others, such as stroke and kidney failure [2].

Sedentarism is the main modifiable risk factor for hypertension development [3]. In opposite, a physically active lifestyle is the best-established non-pharmacological countermeasure to reduce the risk of cardiovascular diseases [4]. According to a reference guideline [5], to be considered physically active adults must perform physical activities for at least 150 min per week of accumulated moderate-intensity or 75 min per week of vigorous-intensity aerobic physical activity (or an equivalent combination of moderate and vigorous activities).

However, sedentarism has a pandemic scale, reaching 28% of adults in the world population [6]. In 2020, the COVID-19 pandemic seems to be increased sedentarism numbers [7], in part due to the adoption of social distancing that suspended many opportunities to exercise, including cardiac rehabilitation services and community health programs [8]. Several position statements have encouraged people to stay active at home, trying to reverse or counterbalance the additional impact of social distance on physical inactivity [9, 10]. Indeed, home-based exercises are considered an alternative for center-based exercise programs so to minimize the discontinuation of regular physical activities.

The effectiveness and safety of exercise training as a frontline non-medication therapy to control blood pressure is well-establish in the literature [11]. Aerobic exercise training has an independent antihypertensive effect that could be added by antihypertensive drugs [12, 13]. Post-exercise hypotension is a common acute effect observed after moderate and dynamic exercise, especially in hypertensive patients. This phenomenon describes the blood pressure falls after a single exercise session due to the persistent reduction in vascular resistance that is not completely offset by the increased cardiac output. Among the possible mechanisms, there are (1) the increment in exercise-induced vasodilator substances; and (2) the arterial baroreflex resetting, which reduces peripheral sympathetic nervous activity [13]. This hypotensive effect of exercise can be extended up to 12 h in hypertensive patients, being plausible to consider that accumulated exercise sessions could provoke a long-lasting effect and a chronic reduction in blood pressure basal values [13, 14].

Therefore, home-based exercises that aimed to control blood pressure in hypertension are the focus of this systematic review. The current study aimed to conduct a systematic review of high-quality controlled trials, following the PRISMA recommendations, to compare the effects of different types of home-based exercises in hypertensive patients.

## Main text

### Methods

#### Bibliographic search

The current systematic review was structured according to PRISMA (Preferred Reporting Items for Systematic reviews and Meta-Analyses) [15].

A systematic search was conducted in Medline, LILACS, and EUROPE PMC databases on July 7th, 2020. All trials were selected and confirmed by all authors. There were no restrictions on publication dates nor patient’s age in the papers evaluated. The search strategy included the following terms: (“home exer*” OR “home-based” or “home-based exercise” or “home-based rehabilitation” OR “home-based functional training” OR “at-home exercise” OR “home-based physical activity”) AND (“blood pressure” OR “high blood pressure” OR “arterial hypertension” OR “hypert*” OR “hypertensive adults”). Filters selected were: Clinical Study, Clinical Trial, Clinical Trial Protocol, Clinical Trial, Phase I, Clinical Trial, Phase II, Clinical Trial, Phase III, Clinical Trial, Phase IV, and Randomized Controlled Trial.

#### Inclusion criteria

Only original trials in the English language were included. The population of this study was composed of hypertensive individuals, being classified as hypertensive according to the parameters of the American Heart Association, whose Systolic Pressure value is equal to or higher than 130 mmHg and the Diastolic Pressure value is equal to or higher than 90 mmHg [5]. Treated and untreated hypertensive patients, with or without comorbidities, such as diabetes, hypercholesterolemia, stroke, previous history of smoking, transient ischemic attack, and acute myocardial infarction, were included. Furthermore, the subjects assigned to the present study were submitted to an intervention based on exercises at home and the FITT principle [16]. Thus, the principal measure is the change on blood pressure after a home-based intervention. Research articles not written in English, review articles, and studies in which intervention was not based on exercise at home were excluded.

## Results

### Literature research

**Fig. 1:**
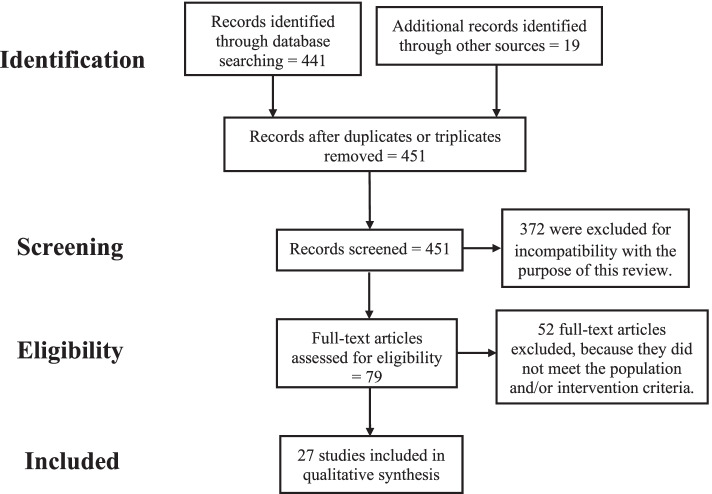
Flowchart of the systematic review process according to PRISMA model [18]

According to pre-established criteria, a total of 441 articles were identified through database searching (Medline = 167; EUROPE PMC = 246; and LILACS = 28), 19 from other sources (study’s references), but 9 were duplicates, remaining 451 articles. After screening the titles, abstracts, and references, 372 were excluded as they did not meet inclusion criteria. Of the remaining 79 eligible full-text articles, and 52 were removed because they did not meet population and/or intervention criteria. Finally, 27 articles were included in the qualitative analysis (Fig. 1). Besides, studies included in qualitative synthesis were evaluated from their risk of bias by each author independently employing the McMaster clinical review form [17]. The consensus was obtained in a later meeting. The results are described in Table 1.

**Table 1:** Risk of bias from studies included in qualitative synthesis

Author, year	Purp	Lit	Study Design			Sample		Outcome		Intervention			Results and Statistical analysis				Con	Total (/17)
			**I**	**II**	**III**	**IV**	**V**	**VI**	**VII**	**VIII**	**IX**	**X**	**XI**	**XII**	**XIII**	**XIV**		
Coghill and Cooper, 2008 [19]	1	1	1	1	1	1	1	1	1	1	0	1	1	1	1	1	1	16
Hua, 2009 [20]	1	1	1	1	1	1	0	1	1	1	0	1	1	1	1	0	1	14
Suter et al., 1990 [21]	1	1	1	1	1	1	1	1	1	1	0	1	1	0	1	1	1	14
Staffileno et al. 2007 [22]	1	1	1	1	1	1	1	1	1	1	1	1	1	0	1	1	1	16
Farinatti et al., 2005 [23]	1	1	1	0	1	1	0	1	1	1	0	0	1	1	1	0	1	12
Farinatti et al., 2016 [24]	1	1	1	1	1	1	0	1	1	1	0	1	1	1	1	1	1	15
Blackwell et al., 2017 [25]	1	1	0	1	1	1	0	1	1	1	0	0	1	1	1	0	1	15
Punia and Kulandaivelan, 2020 [26]	1	1	1	1	1	1	1	1	1	1	1	1	1	0	1	0	1	15
Gordon et al., 2018 [27]	1	1	1	1	1	1	0	1	1	1	0	1	1	1	1	0	1	14
Taylor et al., 2019 [28]	1	1	1	1	1	1	1	1	1	1	0	1	1	1	1	1	1	16
McCaffrey et al., 2005 [29]	1	1	1	1	1	1	0	1	1	1	0	1	1	1	1	1	1	15
Wolff et al. 2013 [30]	1	1	1	1	1	1	1	1	1	1	0	1	1	1	1	0	1	15
Wolff et al., 2016 [31]	1	1	1	1	1	1	1	1	1	1	1	1	1	1	0	1	1	16
Sujatha and Judie, 2014 [32]	1	1	1	1	1	1	0	1	1	1	0	1	1	1	1	1	1	15
Schein et al. 2001 [33]	1	1	1	1	1	1	1	1	1	1	1	1	1	1	1	1	1	17
Viskoper et al. 2003 [34]	1	1	0	0	1	1	0	1	1	1	0	1	1	0	1	0	1	11
Logtenberg et al. 2007 [35]	1	1	1	1	1	1	1	1	1	1	1	1	1	0	1	1	1	16
Anderson et al., 2010 [36]	1	1	1	1	1	1	0	1	1	1	0	1	1	0	1	0	1	13
Meles, 2004 [18]	1	1	1	0	1	1	1	1	1	1	1	1	1	0	1	1	1	16
Rosenthal et al., 2001 [37]	1	1	0	0	1	1	0	1	1	1	0	1	1	0	1	0	1	11
Elliot et al. 2004 [38]	1	1	1	1	1	1	1	1	1	1	1	1	1	0	1	0	1	15
Schein et al. 2009 [39]	1	1	1	1	1	1	1	1	1	1	1	1	1	0	1	1	1	16
Grossman et al. 2001 [40]	1	1	1	1	1	1	1	1	1	1	0	1	1	0	1	1	1	15
Ublosakka-Jones et al., 2018 [41]	1	1	1	1	1	1	0	1	1	1	1	1	1	1	1	1	1	16
Jones et al., 2015 [42]	1	1	1	1	1	1	0	1	1	1	0	1	1	1	1	0	1	14
Jones et al., 2010 [43]	1	1	1	1	1	1	1	1	1	1	1	1	1	1	1	1	1	17
Sangthong et al., 2016 [44]	1	1	1	1	1	1	0	1	1	1	1	1	1	1	1	1	1	16

**I:** Controlled; **II:** Randomized; **III:** Before and after **IV:** Described; **V:** Size justified; **VI:** Reliable **VII:** Valid; **VIII:** Describe in details; I**X:** Contamination avoided; **X:** Co-intervention avoided; **XI:** Reported statistical significance; **XII:** Analysis appropriate; **XIII:** Clinical importance reported; **XIV:** Drop-outs. **1** = Yes**; 0** = No; **Purp.:** Study purpose; **Lit.:** Literature background. **Con.:** Clear conclusions. **N/A:** not applied

Table 2 presents endurance and/or isometric exercise programs. Endurance training was performed from low to moderate [19, 20], moderate to vigorous intensity [22], vigorous [21, 23, 24] and high intensity interval training (HIIT) [25] according to American College of Sports Medicine guideline [16]. Endurance training duration ranged from three to five days a week during four weeks to sixteen months.

**Table 2:** Qualitative synthesis of clinical trials from aerobic and strength training included in the systematic review

Author, year	Sample	Interventions (F.I.T.T)	BP at baseline	Outcomes
Coghill and Cooper, 2008 [19]	EXP: 38♂; 54.8 ± 5 yrs CTL: 29♂; 55.6 ± 4.7 yrs	F: At least 5 days/wk for 12 wks; I: RPE of 12–14; T: at least 30 min T: Walk Briskly	SBP 138 ± 16; DBP 90 ± 10 mmHg	↓SBP; ↔ Resting DBP; ↓BMI; ↓BF; ↓Waist-hip-ratio
Hua, 2009 [20]	EXP: 10♂ 10♀; ♂55.8 + 9.5 yrs; ♀56.3 + 9.6 yrs CTL: 10♂ 10♀; ♂55.9 + 10.2 yrs; ♀58.5 + 11.3 yrs	F: 4 days/wk for 12 wks; I: 35–40% HR reserve and RPE 11–13; T: 4.8 km/day by the end of 12 weeks T: walking	Men SBP 140 ± 11; DBP 92 ± 7 mmHg Women SBP 141 ± 16; DBP 87 ± 9 mmHg	↓SBP and DBP; ↔ HR
Suter et al., 1990 [21]	EXP: 39 ♂; 38.8 ± 8.9 yrs CTL: 22 ♂; 35.2 ± 7.3 yrs	F: 2–6 sessions (self-managed) for 4 months I: 75–86% of HRmax T: at least 120 min per wk T: jogging or walking/jogging	SBP 134 ± 15; DBP 89 ± 11 mmHg	↔ SBP and DBP; ↓Waist-hip-ratio; ↓BMI; ↑endurance capacity
Staffileno et al. 2007 [22]	EXP: 13 ♀; 38.6 ± 5 yrs CTL: 10 ♀; 40.2 ± 6.1 yrs	F: 2–3 sessions/day for 8 wks I: 50–60% HRR T: 10 min/session; 150 min/wk T: lifestyle physical activity (e.g., walking, stair climbing)	SBP 136 ± 7; DBP 91 ± 5 mmHg	↓SBP ↔ resting DBP
Farinatti et al., 2005 [23]	EXP: 26♂ 52♀; 52 ± 12 yrs CTL: 9♂ 7♀; 48 ± 9 yrs	F: 3 days/wk for 16 wks I: 60–80% maximum HR for the age T: 30 min T: Aerobic activity and flexibility exercises	Not reported	↓SBP and DBP; ↓Weight; ↓WHR; ↓ SM; ↓ %BF; ↑TF
Farinatti et al., 2016 [24]	EXP: 7♂ 22♀; 53 ± 11 yrs CTL: 5♂ 9♀; 48 ± 5 yrs	F: 3 days/week for 16 months I: 60–85% HRmax (220 – age) T:30 min T: walking and stretching exercises	SBP 141 ± 20; DBP 85 ± 8 mmHg	↓SBP, DBP and MBP ↓COL; ↑HDL; ↓TRI; ↓BMI; ↓waist circumference; %BF, ↑TF
Blackwell et al., 2017 [25]	EXP H-HIIT: 6♂♀; 52.2 ± 2 yrs EXP H-IHGT: 6♂♀; 51.5 ± 2.3 yrs	H-HIIT: F: 3 days/wk for 4 wks; I: max of repetitions with HR over 85% (HRmax [220 – age]) T: 2 min warm-up + 5 × 1 min of equipment-free T: HIIT (star-jumps, squat thrusts, and static sprints) H-IHGT: F: 3 days/wk for 4 wks I: 30% MVC and HR over 85% (HRmax [220 – age]) T: 4 × 2 min T: isometric handgrip exercise	H-HIIT: SBP 130 ± 5; DBP 81 ± 5 mmHg H-IHGT: SBP 138 ± 4; DBP 93 ± 3 mmHg	H-HIIT: ↔ SBP; ↔ DBP; ↑AT; ↑VO_2max_ H-IHGT: ↓SBP; ↔ DBP; ↔ AT; ↔ VO_2max_
Punia and Kulandaivelan, 2020 [26]	EXP: 10♂ 10♀; 30–45 yrs; CTL: 10♂ 10♀; 30–45 yrs	F: 3 days/wk for 8 wks I: 30% MVC T: 4 × 2 min T: isometric handgrip exercise	SBP 144 ± 8; DBP 93 ± 5 mmHg	↓SBP; ↓DBP; ↓MBP; ↓HR; ↔ PP
Gordon et al., 2018 [27]	EXP: 2♂ 7♀; 47 ± 12 yrs CTL: 2♂ 3♀; 47 ± 9 yrs	F: 3 days/wk for 12 wks I: 30% MVC T: 4 × 2 min T: isometric handgrip training	SBP 137.7 ± 4.1; DBP 88.4 ± 0.8 mmHg	↔ SBP and DBP
Taylor et al., 2018 [28]	EXP: 24♂; 30–65 yrs; CTL: 24♂; 30–65 yrs	F: 3 days/wk for 4 wks I: compatible HR from isometric exercise test T: 4 × 2 min T: isometric wall squat exercise	SBP 137 ± 11; DBP 78 ± 7 mmHg	↓SBP; ↓DBP; ↓PP; ↔ HR; ↑SV and CO at rest; ↓TPR at rest; ↓LF/HF and ↓LFn at rest; ↑HFn, ↑PSD and ↑BRS at rest

*EXP* Experimental group, *CTL* Control group, *H-IHGT* Home-Isometric Hand-Grip Training, *BMI* Body mass index, *BF* Body fat, *COL* Total cholesterol, *HDL* HDL cholesterol, *TRI* Triglycerides, *MVC* Maximal Voluntary Contraction, *wk* Week, *wks* Weeks, Min Minutes, *yrs* Years, *max* Maximum, *HRR* Heart Rate Reserve, *F.I.T.T.* Frequency, intensity, time, and type of exercise, *TPR* Total peripheral resistance, *BP* Blood pressure, *SBP* Systolic Blood pressure, *DBP* Diastolic Blood Pressure, *MBP* Mean Blood pressure, *HR* Heart Rate, *PP* Pulse Pressure, *PSD* R–R power spectral density, *HFn* High frequency R-R in normalized units (%), *LFn* Low frequency R-R in normalized units (%), *LF/HF* Symphato-vagal balance, *BRS* Spontaneous baroreflex sensitivity, *RPE* Rate of perceived exertion, *HIIT* High-Intensity Interval Training, *H-HIIT* Home-High-Intensity Interval Training, *VO*_*2max*_ Maximum oxygen uptake, *AT* Anaerobic threshold, *WHR* Waist-hip measurements, *%BF* Body fat percentage, *SM* Sum of skinfols measurements, *TF* Trunk flexibility, *HRmax* Maximum heart rate, *SV* Stroke volume, *CO* Cardiac output, ↓ decreased, ↑ increased, ↔ unchanged

In three studies, isometric handgrip exercises [26–28] had similar target intensity (30% of maximal voluntary contraction). In one study, isometric wall squat training intensity was controlled by a target heart rate (HR) [45]. Isometric exercise programs were performed three times a week for four to twelve weeks.

Table 3 presents breathing training that includes yoga, device-guided breathing exercises, and slow breathing training with or without inspiratory loading. Yoga programs were composed by breathing and volume-controlled exercises with trunk movements [29–32], device-guided breathing exercises were performed without inspiratory load [18, 33–36], and slow breathing training programs were performed also without load [37–40] or with absolute inspiratory resistive loading (IRL) [18–20 cmH2O] [42–44] or relative IRL defined as 25% of the maximum inspiratory pressure [41]. Regarding training volume, yoga was performed from two days a week to twice-daily sessions (15 min) for 8 to 12 weeks. Device-guided breathing exercises were always performed 7 days/week for 4 to 8 weeks, and slow breathing training (with or without IRL) once or twice daily sessions for 8 weeks.

**Table 3:** Qualitative synthesis of clinical trials from breathing training included in the systematic review

Author, year	Sample	Interventions (F.I.T.T)	BP at baseline	Outcomes
McCaffrey et al., 2005 [29]	EXP: 10♂ 17♀; 56.7 yrs CTL: 9♂ 18♀; 56.2 yrs	F: 3 days/wk for 8 wks I: unloading breathing I: 63 min T: Yoga	SBP 161 ± 10; DBP 98 ± 8 mmHg	↓SBP; ↓DBP; ↓HR; ↓BMI
Wolff et al. 2013 [30]	EXP: 8♂ 20♀; 64 ± 10.3 yrs CTL: 11♂ 16♀; 60.8 ± 11 yrs	F: 7 days/wk for 12wks I: unloading breathing T: 15 min/day T: Yoga	SBP 144 ± 14; DBP 88 ± 6 mmHg	↔ SBP; ↓DBP
Wolff et al., 2016 [31]	EXP: 44♂ 52♀; 64.7 ± 9.2 yrs CTL: 48♂ 47♀; 64.8 ± 7.6 yrs	F: 7 days/wk; 2 sessions/day for 12wks I: unloading breathing T:15 min T: Yoga	SBP 149 ± 12; DBP 88 ± 6 mmHg	↔ SBP; ↔ DBP; Improved self-rated QOL; PSS and HADS
Sujatha and Judie 2014 [32]	EXP: 55♂ 63♀; 30–60 yrs CTL: 55♂ 65♀; 30–60 yrs	F: 5 days/wk for 12wks I: unloading breathing T: 30–45 min T: Hatha Yoga	SBP 153 ± 12; DBP 95 ± 7 mmHg	↓SBP and DBP; ↓HR; ↓BMI ↓Level of stress and anxiety
Schein et al. 2001 [33]	EXP: 18♂ 14♀; 57.8 ± 9.4 yrs CTL: 13♂ 20♀; 56.5 ± 8 yrs	F: 7 days/wk for 8 wks I: unloading breathing T: 10 min T: Device-guided breathing	SBP 157 ± 14; DBP 97 ± 9 mmHg	↓SBP and DBP
Viskoper et al. 2003 [34]	EXP: 10♂ 7♀; 66.5 ± 7.6 yrs	F: 7 days/wk for 8 wks I: Unloading breathing T: 15 min T: Device-guided breathing	SBP 155 ± 10; DBP 89 ± 8 mmHg	↓SBP and DBP; ↓HR
Logtenberg et al. 2007 [35]	EXP:3♂ 12♀; 62.7 ± 6 yrs CTL: 10♂ 5♀; 61.0 ± 7.5 yrs	F: 7 days/wk for 8 wks I: Unloading breathing T: 10 min T: Device-guided breathing	SBP: 154 ± 8; DBP 83 ± 6.7 mmHg	↓SBP and DBP
Anderson et al., 2010 [36]	EXP: 12♂ 8♀; 53.4 ± 2.8 yrs CTL: 9♂ 11♀; 52.9 ± 2.8 yrs	F: 7 days/wk for 4 wks I: < 10 breaths/min, and often ≤ 6 breaths/min T: 15 min T: Device-guided breathing	SBP 142 ± 3; DBP 88 ± 2 mmHg	↓MBP; ↓Breathing rate; ↑Tidal volume; ↓PetCO_2_; ↓24-h BP
Meles, 2004 [18]	EXP:25♂ 19♀; 57 ± 9 yrs CTL: 15♂ 11♀; 49 ± 12 yrs	F: 7 days/wk for 8 wks I: Unloading breathing T: 15 min T: Device-guided breathing	SBP 137 ± 12; DBP 83 ± 9 mmHg	↓SBP and DBP; ↓HR
Rosenthal et al., 2001 [37]	EXP: 7♂ 6♀; 50.5 ± 13.9 yrs	F: 7 days/wk for 8 wks I: Lowest breathing rate for each user T: 15 min T: Slow breathing training	SBP 146 ± 15; DBP 85 ± 8 mmHg	↓SBP; ↓DBP
Elliot et al. 2004 [38]	EXP: 89♀♂;59.5 ± 9.6 yrs CTL: 60♀♂; 58.7 ± 10.5 yrs	F: 7 days/wk for 8 wks I: Unloading breathing T: 15 min T: Slow breathing training	SBP 150 ± 8; DBP 85 ± 9 mmHg	↓SBP and ↔ DBP
Schein et al. 2009 [39]	EXP: 20♂ 13♀; 62 ± 9 yrs CTL: 21♂ 12♀; 63 ± 8 yrs	F: 7 days/wk for 8 wks I: Unloading breathing T: 15 min/day T: Slow breathing training	SBP 148 ± 11; DBP 81 ± 9 mmHg	↓SBP and DBP
Grossman et al. 2001 [40]	EXP: 13 ♂ 5♀; 52 ± 12 yrs CTL: 10♂ 5♀; 50 ± 4 yrs	F: 7 days/wk for 8 wks I: Unloading breathing T: 10 min T: Slow breathing training	SBP 160 ± 18; DBP 95 ± 7 mmHg	↓SBP and DBP
Ublosakka-Jones et al., 2018 [41]	EXP: 8♂ 8♀; 66.4 ± 4.2 yrs CTL: 8♂ 8♀; 68.2 ± 4.8 yrs	F: 7 days/wk; 2 sessions/day for 8wks I: 25% MIP and 50% HRR; T: 6 breaths/min for 5 min/session; 60 breaths/day T: Slow breathing training	SBP 141 ± 7; DBP 70 ± 3 mmHg	↓SBP and DBP;↓PP; ↓HR bpm; ↑MIP; ↑SVC; ↑IC; ↑CE; ↑AE
Jones et al., 2015 [42]	EXP Loaded: 10♂♀; 51.4 ± 5.3yrs EXP No Load: 10♂♀; 53.4 ± 4.3yrs CTL: 10♂♀; 50.4 ± 5.4 yrs	F: 7 days/wk; 2 sessions/day for 8wks I: IRL of 20 cmH_2_O I: 30 min T: Slow breathing training F: 7 days/wk; 2 sessions/day for 8wks I: Unloading breathing I: 30 min T: Slow breathing training	IRL group: SBP 137 ± 13; DBP 81 ± 8 mmHg Unloading breathing group: SBP 136 ± 13; DBP 80 ± 6 mmHg	IRL group: ↓SBP and DBP; ↓HR; ↓MBP Unloading breathing group: ↓SBP and DBP; ↓HR; ↓MBP
Jones et al., 2010 [43]	EXP Loaded: 4♂ 6♀; 51 ± 5 yrs EXP No Load: 4♂ 6♀; 53 ± 4yrs CTL: 3♂ 7♀; 50 ± 5 yrs	F: 7 days/wk; 2 sessions/day for 8wks I: IRL of 20 cmH_2_O T: 30 min T: Slow breathing training F: 7 days/wk; 2 sessions/day for 8wks I: Unloading breathing T: 30 min T: Slow breathing training	IRL group: SBP 142 ± 8.9; DBP 87 ± 5.2 mmHg Unloading breathing group: SBP 141 ± 5.9; DBP 85 ± 4.4 mmHg	IRL group: ↓SBP and DBP; ↓HR; ↓PP Unloading breathing group: ↓SBP and DBP; ↓HR; ↓PP
Sangthong et al., 2016 [44]	EXP Load: 4♂ 6♀; 60–70 yrs; EXP No Load: 1♂ 9♀; 60–79 yrs CTL: 3♂ 6♀; 60–74 yrs	F: 7 days/wk; 30 min/day for 8 wks I: IRL of 18 cmH_2_O T: 6 breaths/min T: Slow breathing training F: 7 days/wk; 30 min/day for 8 wks I: Unloading breathing T: 6 breaths/min T: Slow breathing training	IRL group: SBP 144 ± 8.7; DBP 81 ± 6.7 mmHg Unloading breathing group: SBP 141 ± 11.1; DBP 81 ± 6.2 mmHg	IRL group: ↓SBP; ↔ DBP; ↔ HR; ↓PP Unloading breathing group: ↓SBP; ↔ DBP; ↔ HR; ↓PP

*EXP* Experimental group, *CTL* Control group, *BMI* Body mass index, *PetCO*_*2*_ Partial pressure of carbon dioxide, *IRL* Inspiratory resistive loading, *self-rated QOL* World Health Organization Quality of Life Assessment, *PSS* Perceived stress scale, *HADS* Hospital anxiety and depression scale, *wk* week, *wks* weeks, *min* minutes, *yrs* years, *HRR* Heart Rate Reserve, *F.I.T.T.* Frequency, intensity, time, and type of exercise, *BP* Blood pressure, *SBP* Systolic Blood pressure, *DBP* Diastolic Blood Pressure, *MBP* Mean Blood pressure, *HR* Heart Rate, *PP* Pulse Pressure, *MIP* Maximum inspiratory pressure, *SVC* Slow vital capacity, *IC* Inspiratory capacity, *CE* Chest expansion, *AE* Abdominal expansion

↓: decreased; ↑: increased; ↔ : unchanged

All home-based exercises, except four [19, 21, 27, 31], showed as a primary outcome the blood pressure reduction post-intervention, and secondary outcomes improvements in cardiac autonomic modulation and baroreflex sensitivity [28], inspiratory muscle strength [41], lipids profile and body composition [reduced body fat] [23], quality of life [30], and cardiorespiratory fitness [46].

### Risk of bias

The most design used in home-based studies is the randomized-controlled trial with before and after measurements, but some experimental-controlled studies did not perform and/or described the randomization procedures. For all studies, the literature background and purposes were reported. The sample is well described, but the sample size is justified in twelve studies. Home-based interventions were described in detail, and co-interventions had been avoided since groups were not enrolling in any exercise program. However, the follow-up and monitoring of the control group had been not described in most of the studies, increasing the risk of contamination for this group that may influence the studies’ outcomes.

As regards results, in most of the studies, statistical analysis was appropriated and statistical significance was reported. The clinical importance of results was explored, which is expected since blood pressure reduction is often the primary outcome. However, some studies reported drop-outs throughout intervention protocols. Outcomes were reliable and valid, and conclusions were addressed in most of the studies.

## Discussion

The current systematic review extracted qualitative data of 27 original trials screened from 451 identified studies. The major findings are 1) Both endurance, isometric strength, and respiratory home-based exercise programs were efficient to decrease blood pressure in hypertensive patients, but FITT components were different among them; 2) Despite the home-based interventions reduced blood pressure as the primary outcome, underlying mechanisms seem to be distinct; 3) differences in blood pressure values at baseline must be considered as a potential bias of each study’s outcomes.

Review studies demonstrated the safety and the effectiveness of home-based exercises in cardiac rehabilitation [47] and elderly’s falls prevention programs [48]. From the current review, in seven home-based endurance studies, five reduced blood pressure in hypertensive. Blood pressure was reduced in studies with moderate [19, 20], moderate to vigorous [22], and vigorous exercise intensities [23, 24]. However, in one intervention of moderate to vigorous intensity [21] and another with HIIT [25], blood pressure was unchanged. Although exercise intensity is one important factor to obtain the optimal dose–response relationship between exercise training and blood pressure reduction, other FITT components must also be addressed [11, 16]. Notably, aerobic exercise training has a major effect on blood pressure reductions in hypertensive than normotensive population since the magnitude of blood pressure reduction after an aerobic exercise program seems to be dependent on baseline values [49, 50].

As regarding training volume, weekly frequency varied from 3 to 5 days/week, the session duration ranged from 7 (e.g., HIIT protocol) to 30 min (e.g., most of the continuous exercise protocols), and the programs’ durations were between 4 weeks to 16 months. Notwithstanding some differences in methodological approaches, it was possible to identify home-based moderate to vigorous endurance exercise programs, with 30 min average duration per day for 8 weeks to 16 months, to reduce blood pressure in hypertensive patients.

Isometric exercise programs were also performed at home. Among four studies included in this review, three used handgrip training [25–27], and one used isometric wall squat training [28]. In handgrip studies, target intensity (30% of maximal voluntary contraction) and session duration (4 sets of 2 min) were similar. Isometric wall squat training was performed similar to handgrip with session duration (4 sets of 2 min), and intensity was controlled by a target HR. The HR should be compatible at the end of each stage from the isometric exercise test in visit 1 [28]. All home-based isometric exercise programs [25, 26, 28], except one handgrip study [27], showed reductions in blood pressure after interventions with a duration from 4 to 8 weeks (3 day/wk).

The resistance or strength training alone (i.e., without the combination of another training modality) reduced blood pressure in hypertensive and pre-hypertensive adults [51]. Among strength training programs, isometric and dynamic resistance exercises are effective to reduce blood pressure [4]. Isometric training is widely recommended because of its safety, low cost, easy application at home, and is effective in reduced blood pressure in hypertensive subjects [52]. Among the underlying mechanisms, the reduction in sympathetic activity and increase in vagal tone [45], acute improvements in left ventricular function [53], and improved endothelial function [54] are the most common findings.

**Fig. 2:**
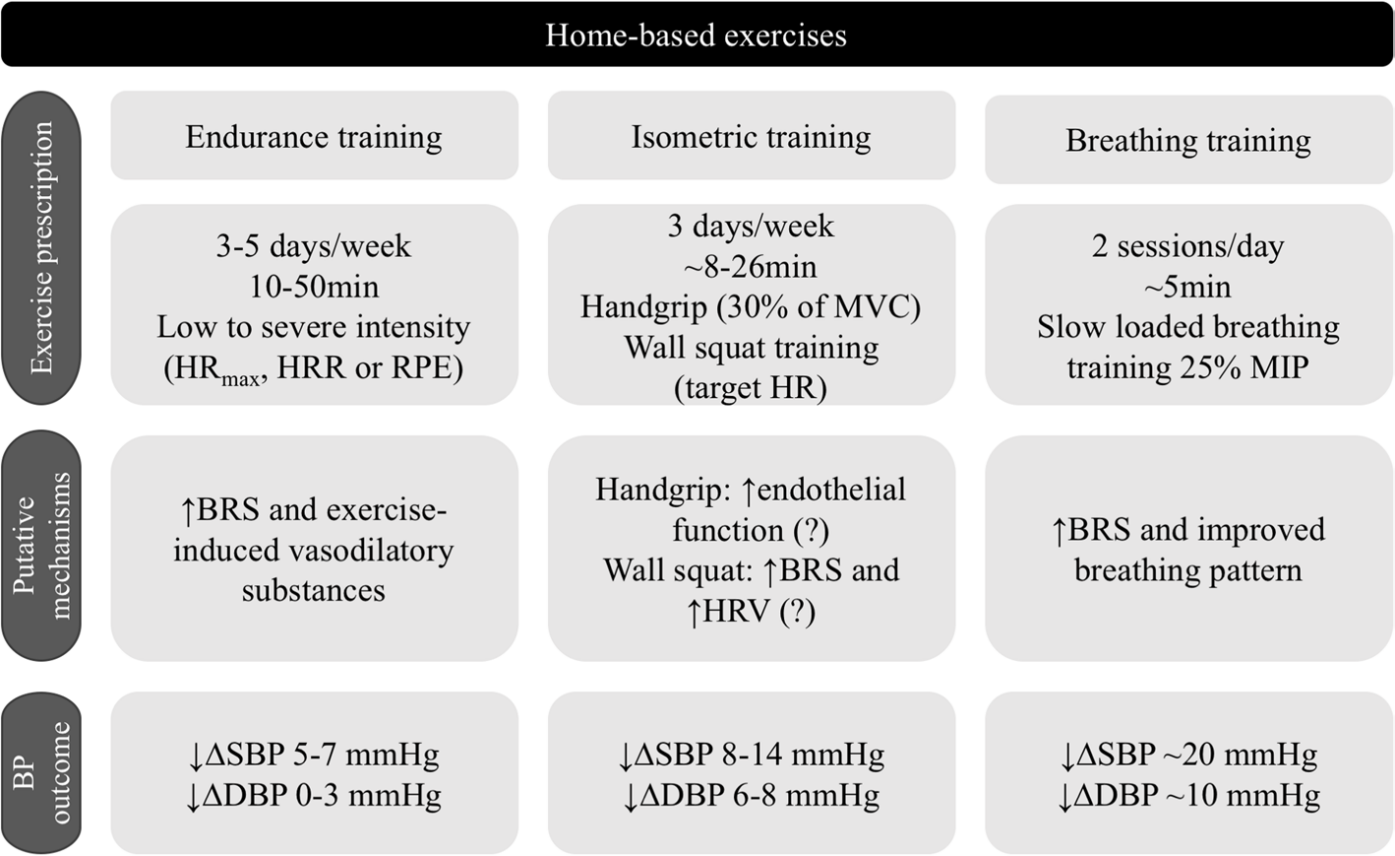
Summary of main effects and putative mechanisms from home-based exercise programs on blood pressure in hypertensive. Target HR should be compatible as the end of each stage from the isometric exercise test in visit 1 [46]. HR, heart rate; HR_max_, maximal heart rate; HRR, heart rate recovery; HRV, heart rate variability; MVC, maximal voluntary contraction; MIP, maximal inspiratory pressure; RPE, relative perception of effort; BRS, baroreflex sensitivity; SBP, systolic blood pressure; DBP, diastolic blood pressure; (?), represents the unknowing mechanisms

Breathing exercises represent most of the home-based programs included in the current systematic review (*n* = 18). Breathing exercises include yoga, device-guided breathing exercises, and slow breathing training with or without inspiratory load. Most of these interventions were performed 5 to 7 days a week, and program duration of 4 to 12 weeks. The intensity was controlled by the exercise’s characteristics as yoga (i.e., breathing and volume-controlled exercises with trunk movements) [29–32], the shortness of breathing frequency as device-guided breathing exercises [18, 31, 33, 34, 36], and slow breathing training with [42–44] and without IRL [37–40]. The sessions’ duration ranged from 10 to 15 min daily, except for two yoga studies when the session lasted 30 to 45 min [32] and 63 min [29]. Except for two studies with yoga exercises [30, 31], all home-based breathing training was effective to reduce blood pressure in hypertensive patients. Interestingly, slow breathing with IRL showed more reduction in blood pressure as compared to isometric and endurance exercise interventions, as shown in Fig. 2.

The well-known effects of breathing on blood pressure regulation supported the development of respiratory exercise programs to reduce high blood pressure in hypertensives. The breathing pattern has a strong influence on heart rate and blood pressure dynamics as described by the cardiorespiratory coupling. A slow breathing pattern [55] or a controlled guided-breathing [56] acutely increased the baroreflex sensitivity and the vagal modulation to the heart [57, 58].

Among respiratory exercises, yoga, controlled breathing with and without loading and guided breathing have demonstrated antihypertensive effects [45, 59]. The Yoga trainee executes slow deep breathing as a combination of low frequency and high tidal volume, presenting higher baroreflex sensitivity and lower hypoxic and hypercapnic chemoreflex responses compared to age-matched controls [57]. In hypertensive subjects practicing yoga, breathing exercises and voluntary control of respiration play an important role in acute and chronic blood pressure management [60]. Controlled guided-breathing has been considered for patients who cannot obtain full control of their hypertension with medical therapy alone or cannot tolerate the adverse effects of the treatment, and is recommended for pre-hypertensive or mildly hypertensive individuals to replace drug prescription [61].

Hypertensive patients may have presented reduced blood pressure through different underlying mechanisms that depend on the home-based exercise protocol (i.e., endurance, isometric, and breathing training). Post-exercise hypotension is a common phenomenon observed in hypertensive patients after endurance exercise, which seems to be explained by two putative mechanisms, increased exercise-induced vasodilatory substances and/or the arterial baroreflex resetting [13]. Therefore, it is plausible to consider that accumulated exercise sessions would provoke a long-lasting effect and a chronic reduction in blood pressure basal values [13, 14, 50]. A recent meta-analysis highlighted that aerobic exercise training improved endothelial function contributing to peripheral vascular resistance and blood pressure reductions. Also, a dose–response relationship between exercise intensity and improved flow-mediated dilation was found [62]. It is particularly important, because hypertensive patients show a reduced nitric oxide bioavailability and vasodilatory capacity, exhibiting an increased vasoconstrictor tone [63]. As regards neural mechanisms, endurance exercise modulates the contributions from the autonomic nervous systems in blood pressure regulation normalizing the sympathetic overactivity observed in hypertension and resetting baroreflex sensitivity [13, 64]. Center-based endurance exercise, 60 min three days per week performed at 70% peak VO_2_ for 4-months, reduced muscle sympathetic nerve activity, improved baroreflex sensitivity and restored blood pressure to normotensive control levels [64]. Besides, home-based endurance training reduced weight, waist-hip-ratio and body fat in hypertensive patients, supporting the reductions in blood pressure [23, 24].

Both endurance and resistance training has been shown to improve baroreflex control as well as vascular function [65]. Otherwise, the putative mechanisms by which isometric exercise training reduces blood pressure in hypertension remain unclear. Cahu Rodrigues et al. [54] demonstrated that 12 weeks of center-based isometric handgrip training improved markers of endothelial function, reducing blood pressure and arterial stiffness in hypertensive patients. Regarding the neural control of the circulation, a study included in the current review [45] showed increased baroreflex sensitivity and reduced sympatho-vagal balance after a home-based isometric wall squat exercise training in hypertensive patients. However, a recent meta-analysis indicates that isometric handgrip training does not improve cardiac autonomic modulation in normotensive as well as in hypertensive subjects [66]. Taken together, some evidence suggests that a low body mass-based isometric training (i.e., handgrip) reduces blood pressure in hypertensive patients due to vascular mechanisms but does not affect neural control of the heart, while a high body mass-based isometric training (i.e., wall squat exercise) improves cardiovascular modulation and reduces blood pressure after a home-based program in hypertensive patients. Little is known about wall squat exercise training, thus the vascular mechanisms, as endothelial function, involved in blood pressure reduction are still to be elucidated.

Among the home-based interventions reviewed in the current study, breathing training have a well-established mechanism that wherefore reduces blood pressure in hypertensive patients. In hypertension, the autonomic imbalance involved in reduced or reset baroreflex sensitivity and chemoreflex induced hyperventilation increases cardiac output, peripheral resistance and blood pressure [67]. The prolonged exhalation in slow or in device-guided breathing exercises, seems to improve baroreflex sensitivity and reduce sympathetic nerve drive and vasoconstriction tone in hypertensive patients. Probably, the activated pulmonary mechanoreceptors that respond to the increased tidal volume (as occurs in slow and deep breathing) act in concert with cardiac mechanoreceptors to inhibit sympathetic outflow to peripheral blood vessels, leading vasodilatation and reducing peripheral resistance and blood pressure [68].

Home-based device-guided breathing training improved the spontaneous breathing pattern at rest in hypertensive patients due to a reduced breathing rate and an increased tidal volume, reducing the blood pressure [36]. Home-based slow breathing training with an IRL also reduced breathing rate and blood pressure in older people with treated and stable isolated systolic hypertension [27]. Finally, a reduction in sympatho-vagal balance and blood pressure was found post-inspiratory muscle training in patients with essential hypertension [67], while an acute IRL increases vagal modulation to the heart in normotensive older women [68]. Taken together, these findings from acute and chronic effects of breathing training, suggest that neural cardiovascular adaptations play a role in blood pressure reductions. Figure 2 summarizes the putative mechanisms in which home-based endurance, isometric and breathing training reduce blood pressure in hypertensive subjects.

In the applicate point of view, all interventions to reduce blood pressure in hypertensive patients could be adapted to a home-based intervention fitting the demand of social isolation. Regarding adherence, home-based exercises could be better than exercises in a sports center or gym. Greater adherence may be explained due to the low-cost characteristic of home-based programs (i.e., without costs with facilities or transportation) and greater flexibility in the participants' routine [9, 10]. Also, obese adults’ enrolled in home-based exercises present greater progress per week when compared to the participants of exercise groups attending a gym. Secondly, home-based exercises are more efficient in a long-term period, but exercise in gym centers remains more effective for short to medium-term benefits [69]. Indeed, both exercise programs (home or centers) improve functional capacity in older adults, but only exercises programs performed three times a week in a fitness center increase strength and cardiorespiratory fitness [70]. Overall, the low adherence in any exercise program seems to involve some concerns as “lack of time” and the accessibility to specific fitness equipment [71, 72]. On the other hand, home-based exercises could increase the accessibility to exercise programs on a large scale and optimize the time expended for physical exercise [47, 73].

## Conclusions

All home-based exercise programs (endurance, isometric strength, and breathing training) included in this current systematic review were effective to reduce blood pressure in hypertensive patients. Despite these encouraging findings, additional randomized controlled trials and mechanistic studies are needed to better provide evidence-based recommendations of home-based exercise programs as antihypertensive therapy.

## Data Availability

Not applicable.

## Abbreviations

FITT: Frequency, intensity, time, and type
HIIT: High intensity interval training
HR: Heart rate
IRL: Inspiratory resistive loading
